# A study of the association between urinary aluminum concentration and pre-clinical findings among aluminum-handling and non-handling workers

**DOI:** 10.1186/s12995-015-0055-8

**Published:** 2015-03-31

**Authors:** Masanori Ogawa, Fujio Kayama

**Affiliations:** Health Service Center, Jichi Medical University, 3311-1, Yakushiji, Shimotsuke Tochigi, 329-0498 Japan; Department of Environmental and Preventive Medicine, Jichi Medical University, 3311-1, Yakushiji, Shimotsuke Tochigi, 329-0498 Japan

**Keywords:** Aluminum, Biological monitoring, Pre-clinical marker

## Abstract

**Background:**

Aluminum is considered to be a relatively safe metal for humans. However, there are some reports that aluminum can be toxic to humans and animals. In order to estimate the toxicity of aluminum with respect to humans, we measured the aluminum concentration in urine of aluminum-handling and non-handling workers and investigated the relationships between their urinary aluminum concentrations and pre-clinical findings.

**Methods:**

Twenty-three healthy aluminum-handling workers and 10 healthy non-aluminum-handling workers participated in this study. Their medical examinations, which were otherwise unremarkable, included the collection of urine and blood. Urinary aluminum levels were analyzed using ICP analysis. As pre-clinical tests, we measured KL-6, SP-D, TRCP-5b, IL-6, and IL-8 in blood and δ-ALA and β2-microglobulin in urine. These were considered to be lung, bone, kidney and inflammation markers. Moreover, we measured 8-OHdG in urine as an oxidative DNA damage marker.

**Results:**

The aluminum concentration in urine ranged from 6.9 to 55.1 μg/g cre (median: 20.1 μg/g cre) in the aluminum-handling workers and from 5.6 to 15.6 μg/g cre (median: 8.8 μg/g cre) in the non-aluminum-handling workers, with a significant difference between them. In the pre-clinical findings, there were no significant differences between these two groups except in the case of δ-ALA. However, there were no significant relationships between aluminum concentration and the pre-clinical findings, work years, age or 8-OHdG in the aluminum-handling workers.

**Conclusions:**

While the excretion of aluminum in urine was elevated in aluminum-handling workers, our findings suggest that low-dose aluminum is not directly harmful to humans, at least when workers’ urinary aluminum concentration is below 55 μg/g cre.

## Background

Aluminum is widely used in everyday life and is considered to be a relatively safe metal for humans compared with other metals such as cadmium, mercury and arsenic, based on the toxicological guideline values of the Joint FAO/WHO Expert Committee on Food Additives (JECFA) [1,2]. Moreover, it is not classifiable as a human carcinogen [3].

However, there have been reports that aluminum is toxic to the lungs and nerves of humans, to the bones in humans who undergo hemodialysis, and to myelopoietic organs in animals. Pulmonary fibrosis has been reported in relation to aluminum exposure [4,5]. Significant changes in neuropsychological tests and dose-dependent central nervous dysfunction have been observed in aluminum-handling workers [6,7]. In patients treated with hemodialysis, aluminum accumulates in the bone with high turnover, and the effects of aluminum on bone, such as osteomalacia, have been found to be dose-dependent and time-dependent [8,9]. Aluminum has been shown to affect δ-aminolevulinic acid dehydratase levels in the blood of mice and the bone marrow of rats [10,11]. Moreover, there are some reports that aluminum nanoparticles affect inflammatory cytokines such as IL-6 and IL-8 and elevate oxygen-reactive species *in vitro* [12,13].

It is important to prevent occupational poisoning before clinical findings appear. Biological monitoring is thought to be a useful tool to detect the effects of exposure on organs before distinct organ damage occurs. However, there are few reports in which the associations between aluminum concentrations in blood or urine and clinical findings have been studied, except for associations with neurological findings.

Various pre-clinical markers show abnormalities before organ damage becomes obvious. Therefore, in order to examine the effect of aluminum on organs, we measured the urinary aluminum concentrations among aluminum-handling and non-handling workers and investigated the relationships between these concentrations and these pre-clinical markers. We focused on the effect of aluminum on lungs, bones, and kidneys and on inflammation and oxidative DNA damage.

Pulmonary fibrosis and neurological findings have been evident under high-dose aluminum exposure [4,14]. Therefore, it is appropriate for pre-clinical markers to be studied in the field under lower aluminum exposure levels.

This study was carried out in a workplace where dust concentration is appropriately controlled under Japanese regulations.

## Methods

### Participants

Twenty-three healthy aluminum-handling workers who were not taking any medications and 10 age-adjusted, healthy non-aluminum-handling workers participated in this study. No significant findings had been made for any of the subjects in the medical examinations, which were carried out annually. Moreover, they had not had any illnesses of the lungs, kidneys, blood or bones. The aluminum-handling workers were engaged in aluminum casting, and the non-handling workers were desk workers at the same facility.

This study was approved by the Ethics Committee of Jichi Medical University. All subjects gave their informed consent.

### Samples

We collected urine and blood samples in examining rooms at a medical facility, not at the aluminum casting factory. The aluminum-handling workers’ blood and urine were collected in the latter half of a work-day in the latter part of the work-week. The non-handling workers’ blood and urine were collected at their convenience.

Aluminum in urine was analyzed using inductively coupled plasma mass spectrometry (ICP-MS). We assigned this analysis to a commissioned company (La Belle Vie, Inc., Tokyo, Japan). This measurement method has a limit of quantification for aluminum in urine of 1.4 ppb.

In order to check the pre-clinical findings, we measured KL-6, SP-D, TRCP-5b, IL-6, and IL-8 in blood and δ-ALA and β2-microglobulin in urine. These markers were intended to indicate lung fibrosis, bone metabolites, inflammation, hematopoietic injury and glomerular damage to the kidney, respectively. These markers were measured by a commercial company (LSI Medience Co., Tokyo, Japan) using their routine method. Moreover, 8-hydroxy-2’-deoxyguanosine (8-OHdG) in urine was measured as an oxidative DNA damage marker using an ELISA kit, the New 8-OHdG Check (Japan Institute for The Control of Aging). Urease treatment was carried out to cancel the effect of urea on the data.

The marker measurements are listed in Table 1.

**Table 1:** **The markers measured in this study**

**Markers**	**Blood**	**Urine**	**Remarks**
Aluminum		○	Measured by inductively coupled plasma analysis
KL-6	○ (serum)		Marker of lung fibrosis
SP-D	○ (serum)		Marker of lung fibrosis
TRACP-5b	○ (serum)		Marker of influence on bone
IL-6	○ (plasma)		Marker of inflammation
IL-8	○ (plasma)		Marker of inflammation
δ-ALA		○	Marker of influence on myelopoietic organ
β2-MG		○	Marker of influence on kidney
8-OHdG		○	Marker of oxidative DNA damage

### Work environmental measurement in the aluminum-handling location

The geometric means of the measurement of aluminum dust in various aluminum-handling locations in the work environment ranged from 0.09–0.31 mg/m^3^. These values fall under Control Class 1 in Japan, which indicates that the workplace is appropriately controlled [15]. Moreover, these values are under the occupational exposure limit of aluminum dust (0.5 mg/m^3^) [16] set by the Japan Society for Occupational Health.

### Statistical analysis

Statistical analyses were performed using Stat View 5.0 (SAS Institute, Inc., Cary, NC, USA). A comparison of each pre-clinical marker and of age between the aluminum-handling workers and non-handling workers was analyzed by the Mann–Whitney *U* test. In the aluminum-handling workers, the relationships between urinary aluminum concentration and each pre-clinical marker, age and work-years were analyzed by Spearman’s rank correlation. The results were considered statistically significant if the p values were <0.05.

## Results

**Figure 1:**
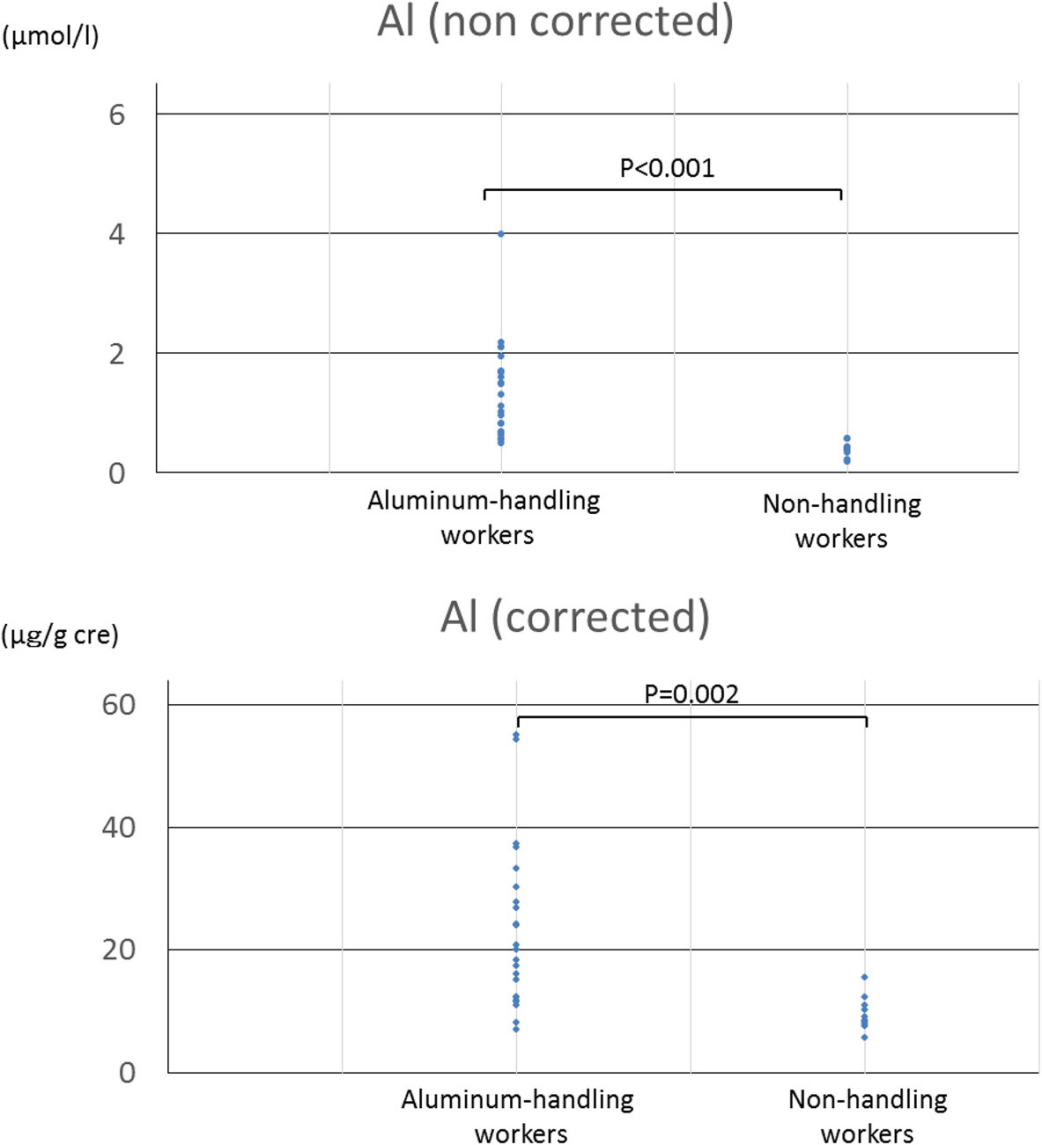
**The distribution of aluminum concentrations in aluminum-handling and non-handling workers.** The upper graph is raw data and the lower is data corrected by urinary creatinine.

The aluminum concentration in urine in the aluminum-handling and non-handling workers are summarized in Table 2, and the distribution of these data are shown in Figure 1. It ranged from 6.9 to 55.1 μg/g cre (median concentration: 20.1 μg/g cre) in the aluminum-handling workers and from 5.6 to 15.6 μg/g cre (median concentration: 8.8 μg/g cre) in the non-handling workers. The difference between aluminum-handling and non-handling workers was statistically significant.

**Table 2:** **Urinary aluminum concentrations in the aluminum-handling and non-handling workers**

	**Aluminum-handling workers**					**Non-handling workers**		
	**Age**	**Al**	**Al**	**Work-year**		**Age**	**Al**	**Al**
	**y.o.**	**μg/g cre**	**μmol/l**	**Year**		**y.o.**	**μg/g cre**	**μmol/l**
	27	6.9	0.62	6		36	5.6	0.33
	38	8.1	1.02	7		54	7.6	0.57
	30	10.9	0.54	2		52	7.8	0.42
	46	11.5	0.48	20		58	8.3	0.41
	48	11.6	0.68	22		34	8.4	0.43
	24	12.3	0.66	5		30	9.1	0.18
	52	12.3	0.81	31		44	10.2	0.39
	55	15.1	0.82	36		40	11.0	0.37
	34	16.1	0.95	15		23	12.3	0.22
	37	17.4	1.49	1		47	15.6	0.56
	19	18.4	1.48	1	Median		8.8	0.40
	43	20.1	1.69	8				
	40	20.7	1.31	21				
	52	24.0	2.10	31				
	29	24.2	2.10	4				
	36	26.8	2.18	17				
	47	27.9	1.70	19				
	33	30.2	1.59	3				
	54	33.4	3.99	35				
	53	36.8	1.12	25				
	54	37.3	0.56	36				
	60	54.4	1.95	31				
	41	55.1	1.68	7				
Median		20.1	1.31

**Figure 2:**
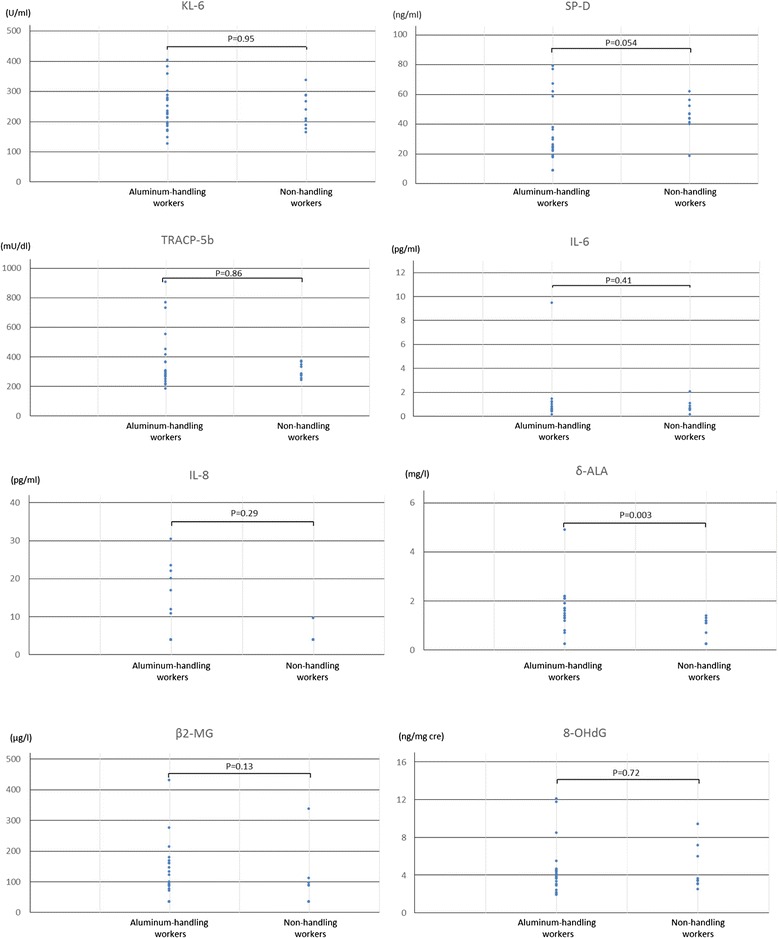
**Distributions of pre-clinical markers in aluminum-handling and non-handling workers.** There were no significant differences in KL-6, SP-D, TRCP-5b, IL-6, IL-8, β2-microglobulin and 8-OHdG between these two groups. A significant difference was found in δ-ALA levels.

In the pre-clinical findings, although there was a significant difference in δ-ALA, there were no significant differences in KL-6, SP-D, TRCP-5b, IL-6, IL-8, β2-microglobulin or 8-OHdG between the two groups (Figure 2). The data under the detection limit were assumed to be half of the detection limit.

In the aluminum-handling workers, no significant relationship was found between aluminum concentration and each pre-clinical marker, work-years or age.

## Discussion

The excretion of aluminum in urine was elevated in aluminum-handling workers. However, there was no significant relationship between aluminum concentration and work-years, age, or any pre-clinical finding or 8-OHdG in this study. From these findings, no health effects of aluminum at urinary concentrations below 55 μg/g cre could be observed.

Threshold levels have been reported for the association between urinary aluminum and neurological effects. Pre-clinical neurotoxic effects are observed when serum aluminum levels exceed 10 μg/l [6]. Serum aluminum levels of 0.25–0.35 μmol/l (6.8–9.5 μg/l), and urinary aluminum levels of 4–6 μmol/l (108–162 μg/l) appear to represent a threshold for observed adverse neurological effects [7]. Iregren et al. [17] indicated that a urinary level of 100 μg/l was a critical concentration for the development of neurological effects. However, there are few reports in which the associations between the aluminum concentration in blood or urine and clinical findings have been studied, except for associations with neurological findings. The urinary aluminum concentration in aluminum-handling workers observed in this study was much lower than the threshold for neurological effects. This is due to the low dust concentration in the air at the workplace studied.

Sjögern et al. [18] reported a difference in urinary aluminum concentrations between welders with 0.08 to 2 years’ exposure and those with 18 to 20 years’ exposure. On the other hand, our study found no relationship between urinary aluminum and work-years. The aluminum concentration in the air in the study by Sjögren et al. was relatively high, at 2.4 mg/m^3^ (8 h-TWA). In our study, the total dust concentration in the air was much lower, at 0.09–0.31 mg/m^3^. All workers in this study have at least 16 hours between shifts. There are reports suggesting that about 1% of aluminum in welding fumes is rapidly absorbed from the lungs [19], and about 45% of that is cleared from the lungs after one day [20]. We suppose that no relationship between urinary aluminum concentration and work-years could be detected because the concentration of inhaled aluminum was not very high and the time interval to the next work shift was relatively long.

Many studies have been done on pulmonary fibrosis in aluminum-handling workers [4,5]. Aluminum induces the impairment of bone formation [21], and this occurs mostly in patients with chronic renal disease who are undergoing renal dialysis [3]. In an *in vitro* study, aluminum nanoparticles were shown to inhibit the inflammation reaction (e.g. IL-6, IL-8) to methicillin-resistant *Staphylococcus aureus* [12]. Dey et al. [13] reported that aluminum nanoparticles shifted into the cells and elevated the level of reactive oxygen species. An animal experiment showed that aluminum has a distinct effect on blood δ-aminolevulinic acid dehydratase (ALA-D) activity according to the aluminum concentration in the blood [10]. No significant differences in KL-6, SP-D, TRCP-5b, IL-6, IL-8, or 8-OHdG were detected between aluminum-handling and non-handling workers; therefore, although the excretion of aluminum in urine was elevated in aluminum-handling workers, this study suggests that aluminum is not harmful to humans. Some metals do cause kidney damage and an elevation of urinary β2 microglobulin [22,23] but no reports have yet shown that aluminum causes renal dysfunction in healthy people. Urinary β2 microglobulin was measured in the present study; however, it was not elevated in aluminum-handling workers compared with non-handling workers.

Some stomach medicines and foods contain aluminum, and the ingestion of these may affect the urinary aluminum concentration. Generally, the aluminum concentration in food is lower than that in aluminum-containing medicine, and the absorption of aluminum from the gastrointestinal tract has been reported to be very low [24,25]. Therefore, we had the participants restrain their intake of aluminum-containing drugs, but we did not limit their food intake. Because of this restriction, we suspected that the elevation of urinary aluminum in the aluminum-handling workers resulted mainly from inhalation.

In this study, there were some smokers among both the aluminum-handling and non-handling workers. Iarmarcovai et al. mentioned that although cadmium was significantly more prevalent in smokers [26], the concentrations of other metals including aluminum did not differ between smokers and non-smokers. Therefore, we did not distinguish between smokers and non-smokers in our analysis.

As for the sampling time, one report found that although urinary aluminum concentrations were not significantly different between the beginning and end of the work-week, the concentration at the end of the week was higher [26]. Therefore, the samples used in this study were collected in the latter half of the work-day in the latter part of the work-week.

The primary objectives of biological monitoring of aluminum are to detect excessive aluminum entering the lungs and to prevent the harmful accumulation of aluminum in target organs. Biological monitoring of aluminum can be effectively used for these purposes [26]. When performing biological monitoring, the determination of aluminum in urine is recommended due to the higher sensitivity and robustness of this measure compared to the measurement of aluminum in plasma [27,28]. This is why urinary aluminum was used to assess the aluminum concentration in our study.

Among the pre-clinical markers, only δ-ALA showed a significant difference between the aluminum-handling and non-handling workers. Lead is well known for inhibiting ALA-D activity and increasing the excretion of δ-ALA into urine [23]. In order to rule out the effect of lead on δ-ALA in aluminum-handling workers, lead in urine was also measured by ICP-MS. There was no significant difference in urinary lead levels between the two groups. Consequently, the elevation of δ-ALA in aluminum-handling workers’ urine may have been due to aluminum. However, the elevation of δ-ALA in urine did not exceed the occupational exposure limits based on biological monitoring issued by the Japan Society for Occupational Health [16], and the level of elevation observed in this study has not shown any health effects.

There are some limitations to this study. First, the aluminum concentration in the air in this particular workplace is low. Thus, the urinary aluminum concentration is lower than that seen in cases with obvious adverse effects. However, it is appropriate for pre-clinical markers to be studied in the field under lower aluminum levels. Second, the sample size may be small. However, the urinary aluminum concentration in aluminum-handling workers was found to be significantly higher than that in non-handling workers. Thus, the effect of aluminum on the pre-clinical markers could be assessed. Since no previous studies have focused on the effect of pre-clinical markers under the aluminum exposure, this study is useful from the viewpoint of occupational health.

From these findings, no health effects of aluminum at urinary concentrations below 55 μg/g cre (4 μmol/l) could be observed. Aluminum has low toxicity to humans; however, it is still important to try to reduce aluminum exposure as much as possible. It has been reported that workers in workplaces without collective protection devices showed significantly higher urinary aluminum concentrations than those in workplaces equipped with smoke-extraction systems [26]. Therefore, equipping workers and workplaces with appropriate collective protective devices, such as local exhaust ventilation, is an effective measure for reducing workers’ exposure to aluminum; wearing personal protective equipment, such as a dust mask, is also recommended.

## Conclusions

The excretion of aluminum in urine was elevated in aluminum-handling workers. Although our sample study was small and the workplace relatively free of aluminium dust, our findings suggest that the aluminum in such a workplace is not directly harmful to the lungs, bones, and kidneys and it does not cause inflammation and oxidative DNA damage in humans, at least when urinary aluminum is below 55 μg/g cre.
